# Ubiquitin-specific protease 47 is associated with vascular calcification in chronic kidney disease by regulating osteogenic transdifferentiation of vascular smooth muscle cells

**DOI:** 10.1080/0886022X.2022.2072337

**Published:** 2022-05-04

**Authors:** Qiong Xiao, Yun Tang, Juhua Xia, Haojun Luo, Meidie Yu, Sipei Chen, Wei Wang, Lei Pu, Li Wang, Guisen Li, Yi Li

**Affiliations:** a Department of Nephrology, Sichuan Provincial People’s Hospital, University of Electronic Science and Technology of China, Chengdu, People’s Republic of China; b Sichuan Clinical Research Center for Kidney Diseases, Clinical Immunology Translational Medicine Key Laboratory of Sichuan Province, School of Medicine, University of Electronic Science and Technology of China, Chengdu, People’s Republic of China; c Chinese Academy of Sciences Sichuan Translational Medicine Research Hospital, Chengdu, People’s Republic of China; d The First Affiliated Hospital of Chongqing Medical and Pharmaceutical College, Chongqing, People’s Republic of China; e Jintang First People’s Hospital, Chengdu, People’s Republic of China

**Keywords:** Chronic kidney disease, label-free quantification, serine/threonine-protein kinase akt-1, ubiquitin-specific protease 47, Vascular calcification, vascular smooth muscle cells, β-transducin repeat-containing protein

## Abstract

Chronic kidney disease (CKD) has recently become a serious health and social concern. Vascular calcification, a common complication of CKD, is a risk factor that increases the incidence and mortality of cardiovascular events in patients with CKD. However, there are currently no effective therapeutic targets that can facilitate treatment with fewer side effects for vascular calcification in CKD. To identify potential therapeutic targets, we performed label-free quantification (LFQ) analyses of protein samples from rat aortic vascular smooth muscle cells (RASMCs) after high-phosphorus treatment by nano-UPLC–MS/MS. We determined that ubiquitin-specific protease 47 (USP47) may be associated with CKD vascular calcification by regulating the osteogenic transdifferentiation of the vascular smooth muscle cell (VSMC) phenotype, thus suggesting a novel and potentially effective therapeutic target for CKD vascular calcification. USP47 knockdown significantly reduced the expression of β-transducin repeat-containing protein (BTRC), serine/threonine-protein kinase akt-1 (AKT1), Klotho, fibroblast growth factor (FGF23), and matrix Gla protein (MGP) in RASMCs after high-phosphorus treatment. Consistent with the results of protein–protein interaction (PPI) analyses, USP47 may be involved in regulating osteogenic transdifferentiation markers, such as runt-related transcription factor 2 (RUNX2), Klotho, FGF23, and MGP through the BTRC/AKT1 pathway upon CKD vascular calcification. These data indicate that USP47 may be associated with vascular calcification in CKD by regulating osteogenic differentiation of VSMCs. USP47 may regulate osteogenic transdifferentiation in VSMCs upon CKD vascular calcification through a process involving the BTRC/AKT1 pathway. This study identified a novel potential therapeutic target for the treatment of vascular calcification in CKD.

## Introduction

Since 1990, there have been approximately 697.5 million CKD patients, and the global all-age prevalence of CKD has increased by 29.3%, while the global all-age mortality rate has increased by 41.5% [1]. In China, the prevalence of CKD has exceeded 10.8% [2]. CKD has become a considerable health and social problem for humans worldwide due to its clinical manifestations and complications. Several serious complications, including atherosclerosis, vascular calcification, CKD-mineral and bone disorder (CKD-MBD), fractures, anemia, hypothyroidism, and severe secondary hyperparathyroidism, are primarily associated with CKD [3–5]. Among these complications, vascular calcification is a vital risk factor that increases the incidence and mortality of cardiovascular events in patients with CKD [6,7]. This complication represents an obvious cardiovascular abnormality and is indicative of a poor outcome with calcification deposition in the smooth muscle layer of the central and peripheral arteries [8–11]. Once believed to be a passive degenerative process, vascular calcification is now considered a complex and regulated process associated with the activation of cellular signaling pathways, calcium and phosphorus metabolism disorders, secondary hyperparathyroidism, circulatory inhibition of calcification, and various genetic factors and hormones [12–15]. However, the pathogenesis of vascular calcification in patients with CKD remains unclear.

Vascular calcification is not only induced by a high-phosphorous and high-calcium milieu but is also precisely regulated by delicate and well-organized biological processes involving a balance between osteochondrogenic signaling and anti-calcification events [16,17]. The major drivers of vascular calcification, such as aging, uremia, mechanical stress, oxidative stress, and inflammation, can facilitate osteogenic differentiation of VSMCs in patients with CKD [18,19]. Due to the complicated pathological mechanism, effective therapeutic targets that can facilitate treatment with fewer side effects for vascular calcification in patients with CKD are still needed. To identify potential therapeutic targets for CKD vascular calcification, we performed LFQ using nano-UPLC–MS/MS analyses of protein samples from RASMCs following high phosphorus treatment.

USP47 is a deubiquitinase that physiological functions and enzymatic properties remain unclear in the ubiquitin specific protease family. USP47 is considered an attractive antagonist for cancer therapy [20–22]. Meanwhile, other studies suggest a link between USP47 and viruses [23,24]. USP47 also plays important roles in MAPK [25] and Wnt signaling [26]. However, no studies have investigated the association between USP47 and vascular calcification in CKD. We hypothesized that USP47 may be associated with CKD vascular calcification by regulating osteogenic transdifferentiation of the VSMC phenotype, thus suggesting a novel and potentially effective therapeutic target for patients with CKD vascular calcification.

## Materials and methods

### Reagents and antibodies

Rabbit polyclonal anti-transgelin (SM22α) (10493-1-AP), rabbit polyclonal anti-tumor necrosis factor ligand superfamily member 11 (RANKL) (23408-1-AP), rabbit polyclonal anti-RUNX2 (20700-1-AP), and HRP-conjugated β-actin mouse monoclonal antibody (HRP-60008) were all obtained from Proteintech (China). Rabbit polyclonal anti-RUNX2 (860139), rabbit polyclonal anti-AKT1 (380617), and HRP-conjugated goat anti-rabbit IgG secondary antibodies (511203) were all purchased from ZEN BIO (China). Rabbit polyclonal anti-USP47 (NBP1-85942) was purchased from Novus (USA). Rabbit polyclonal anti-BTRC antibody (abs136054) was purchased from ABsin Bioscience (China). USP47 siRNA (siB170517035505-1-5), negative siRNA (siN0000001-1-5), and liposome reagent (RiboFECT CP) were purchased from RIBOBIO (China). Immobilon western chemiluminescent HRP substrate (Wbkls0500) was purchased from Millipore (USA), and TRIzol reagent (15596026) was purchased from Life Technologies (USA).

### Cell cultures and transfections

RASMCs were obtained from the National Collection of Authenticated Cell Culture (#GNR7, China). The cells were cultured in Dulbecco’s modified Eagle’s medium supplemented with 10% fetal bovine serum and antibiotics containing 100 U/mL of penicillin and 100 U/mL of streptomycin. To mimic calcification in RASMCs, 10 mM β-glycerophosphate was used to treat RASMCs for 72 h [27,28]. RASMCs were treated with modified Dulbecco’s modified Eagle’s medium without β-glycerophosphate, as a normal control. Cells were cultured at 37 °C in an atmosphere containing 5% CO_2_. To observe the role of USP47 in calcification, RASMCs were seeded at a density of 2 × 10^5^ cells/well. After overnight adhesion, the cells were transfected with 50 nM USP47 siRNA or negative siRNA using liposome reagent for 24 h.

### Study participants and data collection

For human tissue sample collection, four arterial tissue samples were collected, including two from the radial artery of chronic hemodialysis patients who presented for arterial-venous fistula repair and two from the lower extremity artery of patients who presented for amputation due to severe motor accidents and had normal renal function. To observe USP47 expression in vascular calcification, maintenance hemodialysis (HD) patients aged 18–80 years who had been undergoing regular hemodialysis for at least 3 months from January 2018 to December 2020 at Sichuan Provincial People's Hospital were included with full ethical approval from the Sichuan Academy of Medical Sciences and the Sichuan Provincial People’s Hospital Medical Ethics Committee (No. 2017.36). All maintenance HD patients were confirmed to have vascular calcification according to a coronary artery calcification score of ≥30 Agatston units [29]. Exclusion criteria included: (1) unwillingness or inability to complete the research process; (2) participation in other intervention studies; (3) complications with severe systemic diseases or severe wasting diseases such as severe malnutrition, cirrhosis, and tumors, such as multiple myeloma; (4) hospitalization due to acute disease arising during the research process; (5) undergoing peritoneal dialysis; and (6) received corticosteroids or immunosuppressants for the past six months. Healthy subjects who underwent routine health checks at the same hospital during the same period were enrolled as controls. Clinical characteristics were recorded, including age (years) and sex for all subjects, and vintage (months) and comorbidities for HD patients were also assessed. The study was approved by the Institutional Review Board of the Sichuan Provincial People’s Hospital (No. 2017. 36) and was conducted in compliance with the principles of the Helsinki Declaration. Written informed consent was obtained from all participants prior to the study.

### ELISA

Fasting serum samples were collected for laboratory analysis. For HD subjects, serum samples were obtained immediately before the first HD session of the week. Serum samples were collected after centrifugation for 15 min at 3000 rpm at 4 °C and stored at −80 °C within 30 min of sampling. Biochemical tests for creatinine, serum phosphorus, serum calcium, parathyroid hormone, alkaline phosphatase, and FGF23 were performed at the central laboratory of the Sichuan Provincial People’s Hospital. Human USP47 levels were measured using an ELISA kit (ZC-54646, ZCI BIO, China), according to the manufacturer's instructions. The optical density of each sample was determined within 5 min using a microplate reader (Model 680, BIO-RAD, USA) at 450 nm.

### Label-free quantification

LFQ was performed using protein sample preparation and nano-UPLC-MS/MS analysis. RASMCs were lysed in RIPA buffer containing 1% phenylmethanesulfonyl fluoride, 1% sodium deoxycholate, 1% NP-40, and 1% SDS. The protein concentration of each sample was calculated using the Pierce BCA Protein Assay Kit (Thermo Scientific, USA), with crystalline bovine serum albumin as the calibration standard. For acetone precipitation, 100 μg of protein was diluted to 1 mg/mL for each sample. Ammonium bicarbonate (100 mM) was added to 100 μL of 1% sodium deoxycholate, and the mixture was sonicated for 15 min in a water bath to dissolve the protein pellet. To reduce disulfide bonds, 5 mM Tris (2-carboxyethyl) phosphine was added to each sample for 10 min at 55 °C. The sample was alkylated with 10 mM iodoacetamide for 15 min in the dark at room temperature. The sample was digested with 2 µg of trypsin (Promega, USA) overnight at 37 °C. Sodium deoxycholate was removed from the protein samples using trifluoroacetic acid. To desalt the peptide, we equilibrated a C_18_ column with 500 μL acetonitrile, washed the column with 1000 μL 0.1% formic acid, eluted the peptide with 400 μL 70% acetonitrile, and then resuspended the peptide in 0.1% formic acid for LC–MS/MS analysis.

For each test, 2 µg of the digested protein sample was separated and analyzed using a Nano-UPLC EASY-nLC1200 (Thermo Scientific, USA) coupled with a Q-Exactive mass spectrometer (Thermo Finnigan, USA) without any discontinuation. The instrument was connected to a 100 μm i.d. × 15 cm Reprosil-Pur 120 C18-AQ column with a particle size of 1.9 μm. The following LC buffers were used: buffer A (0.1% FA with 2% ACN) and buffer B (0.1% FA with 80% ACN). Peptides were eluted at a flow rate of 300 nL/min for 120 min. Gradient B was performed at 5% for 3 min, 8–35% for 92 min, 35–45% for 20 min, 45–100% for 2 min, 100% for 2 min, 100–2% for 2 min, and 2% for 2 min. The peptides were then analyzed in data-dependent MS/MS mode with the following settings: a resolution of 70,000 for MS1 and 17,500 for MS2. The MS scan range was 350–1600 *m*/*z*. The automatic gain control target was set to 3 × 10^6^ counts for MS1, whereas the MS2 AGC target was set to 1 × 10^5^. The dynamic exclusion time window was set to 40 s. The isolation window was set to 2 *m*/*z*.

### Qualitative analysis of proteomic data

After each LC–MS/MS test, the raw MS files were processed using MaxQuant (version 1.5.6.0). The protein sequence database was searched using UNIPROT software. This database and its reverse decoy were then searched using MaxQuant software. The analysis was based on the LFQ intensities and standard deviation of this value for all experimental groups. Trypsin was used as a specific enzyme and three missed cleavages were allowed. Carbamidomethyl was used as a fixed modification. Oxidation M and acetyl protein N-terms were considered variable modifications. Both peptide and protein FDR should be less than 1%. Only unmodified and unique peptides were used for quantification. All other parameters were reserved as defaults. The missing values were calculated to replace random numbers selected from a normal distribution using Perseus software (Version 1.4.1.3, Germany). Protein groups with non-missing values less than those of the replicates were discarded. Proteins were defined as possessing significant differences by comparing the mean LFQ intensities with a fold change minimum of ±2 (*p* < 0.05) at the protein level.

### Bioinformatics analysis

Bioinformatics analysis was conducted using the Search Tool for the Retrieval of Interacting Genes/Proteins (STRING) database to evaluate protein-protein interactions. The interactions provided by STRING were primarily based on the confidence score and other collateral information, such as the provided protein domains and 3 D structures. The thickness of the line indicates the strength of the interaction between the proteins.

### Animals

Male wildtype Wistar rats (8–10 weeks of age; DOSSY, China) were used for experiments. The rats were randomly assigned to two groups: the normal control group (*n* = 7) and the CKD vascular calcification group (*n* = 7). Rats in the CKD vascular calcification group were fed with 0.75% adenine diet (PHR1383, Sigma, USA), were injected intraperitoneally with 3 × 10^6^ U vitamin D3 (V8070, Solarbio, China), and 30 min later were given intragastrically 6.25 mg/kg nicotine (SN8140, Solarbio, China) once a day [27]. After 12 weeks of treatment, the rats were anesthetized with 1% pentobarbital sodium (11715, Sigma, USA) at 4 mL/kg to collect blood samples and aortic and renal tissue samples. All animal experiments were performed in accordance with the ethical standards of the Center of Animal Experiments of the Sichuan Academy of Medical Sciences and Sichuan Provincial People’s Hospital (No. 2017. 36).

### Alizarin red S staining

Arterial tissue was fixed in 10% formalin, dehydrated, embedded, and then incubated with Alizarin Red S stain (G8550, Solarbio, China) to detect calcium deposition. For alizarin red S staining, the slices were treated with 2% alizarin red S solution for 5 min, McGee-Russell reagent for 10 s, hematoxylin for 2 min, and followed with multiple ddH_2_O washes. The slices were sealed with neutral gum and imaged under a microscope.

### Von kossa staining

To identify calcification, von kossa staining was performed according to the manufacturer’s instructions (G3282, Solarbio Life Science, China). Briefly, the cells were removed from the medium in a six-well plate, washed three times with saline solution, fixed for 30 min in 10% formalin, and rinsed three times with ddH_2_O. The cells were then incubated with 5% silver nitrate solution at ambient temperature for 10 min under ultraviolet light until the color developed. The silver nitrate solution was discarded, and the cells were washed with ddH_2_O for 3 min. Under light, the cells were treated with 3% sodium thiosulfate solution for 2 min and rinsed for 5 min. After hematoxylin staining for 5 min and treatment with 1% hydrochloric acid alcohol solution for 10 s, the slides were rinsed until they became blue. The dried coverslips were sealed with neutral gum. Finally, the slides were imaged under a microscope.

### Real-time PCR

Real-time PCR was conducted using a two-step method to identify the differential expression of USP47 within the cells. Cells were collected and total RNA was extracted using TRIzol reagent. cDNA was synthesized using the PrimeScript RT Reagent Kit (RR047A, TaKaRa, China). Genomic DNA was removed from the RNA sample prior to cDNA synthesis, and the total volume of the reverse transcription reaction was 20 μL. The solution contained 10 μL of Master Mix, 1 μL of PrimeScript RT Enzyme Mix I, 1 μL of RT Primer Mix, 4 μL of 5 × PrimeScript Buffer, and 4 μL of RNase-Free dH_2_O. The reactions were run on a GeneAmp PCR System (9700, ABI, USA) at 37 °C for 15 min, 85 °C for 5 s, and maintained at 4 °C. A TB Green Premix Ex Taq II kit (RR820A, TaKaRa, China) was used to prepare the PCR reaction solution. The total volume of the reaction solution was 25 μL, including 1 μL of forward and reverse primers, 2 μL of cDNA diluted five-fold, 12.5 μL of TB Green Premix Ex Taq II, and deionized water (DNase/RNase-Free, TIANGEN, China). PCR reactions were conducted in a CFX96 Real-Time PCR Detection System (CFX96 Touch, Bio-Rad, USA) with the following cycling conditions: one cycle at 95 °C for 30 s, 40 cycles at 95 °C for 5 s, and 60 °C for 30 s. The primer sequences were as follows: GAPDH-F, AGTGCCAGCCTCGTCTCATA; GAPDH-R, GATGGTGATGGGTTTCCCGT; USP47-F, GATGTGATTCCCTTGGATTGCT; USP47-R, AACCCCATTGGTGTATCTTCTTC; FGF23-F, CACTACCTGGTGAGCTTGGG; FGF23-R, CTTCCTCTGCACTCGGTAGC; Klotho-F, GACTTCGTGCTAGGCTGGTT; Klotho-R, AGCTCAAGGTTGGTCCGAAG; MGP-2, CGCCTACAACCGCTACTTCA; and MGP-2, CAAGCAACGCACACGAATCT (TSINGKE Biological Technology, China).

### Immunohistochemistry

Immunohistochemical staining was performed on 3 μm sections of paraffin-embedded artery tissue samples to detect the protein expression levels of USP47. Dewaxed and rehydrated tissue sections were incubated with 3% hydrogen peroxide for 10 min. Heat-induced antigen retrieval was performed using the 5 mM citrate buffer (MVS-0101, MXB, China) in a pressure cooker for 15 min. The retrieval was performed at pH of 8.0. The slides were incubated with the USP47 antibody at a dilution of 1:20 overnight at 4 °C. The secondary antibody anti-rabbit IgG polymer (K5007, Envision, Denmark) was used for 45 min at room temperature. Images were captured using a digital microscope slide scanner (Pannoramic MIDI, 3DHISTECH, Hungary).

### Western blot analysis

Following treatment, the cells were collected. Whole-cell lysates were prepared by lysing cells in RIPA buffer (20 mM Tris, pH 7.4, 150 mM NaCl, 1 mM EDTA, 1% Nonident P-40, and 0.1% SDS) containing the complete protease inhibitor phenylmethanesulfonyl fluoride (ST506, Beyotime, China). Proteins were separated by 10% or 12.5% SDS-polyacrylamide gel electrophoresis and transferred to Immobilon-P polyvinylidene difluoride membranes (Millipore, USA). The membranes were incubated successively with 5% nonfat milk in Tris buffer (20 mM Tris-HCL and 150 mM NaCl, pH 7.5) and 0.05% Tween 20 for 2 h and with primary antibodies as follows: anti-RANKL, anti-RUNX2, anti-USP47, anti-transgelin/SM22α, anti-AKT1, and anti-BTRC at a dilution ratio of 1:1000 at 4 °C overnight. For the anti-β-actin antibody, the dilution ratio was 1:10,000. The blots were then incubated with horseradish peroxidase-conjugated goat anti-rabbit secondary antibody at a dilution ratio of 1:10,000 for 2 h. The signal was detected using a Fusion FX7 imaging system (FX7, Vilber Lourmat, France).

### Statistical analysis

GraphPad Prism software was used for statistical analysis (version 7.0, GraphPad Software, USA). Values are expressed as the means ± standard deviation (SD). The differences among multiple groups were evaluated using one-way analysis of variance (ANOVA) and between two groups using the Student’s unpaired *t*-test analysis. Correlation analysis was conducted using Pearson Correlation. A value of *p* < 0.05 determined the threshold for statistical significance.

## Results

### High phosphorus concentrations facilitated RASMC calcification

**Figure 1.**
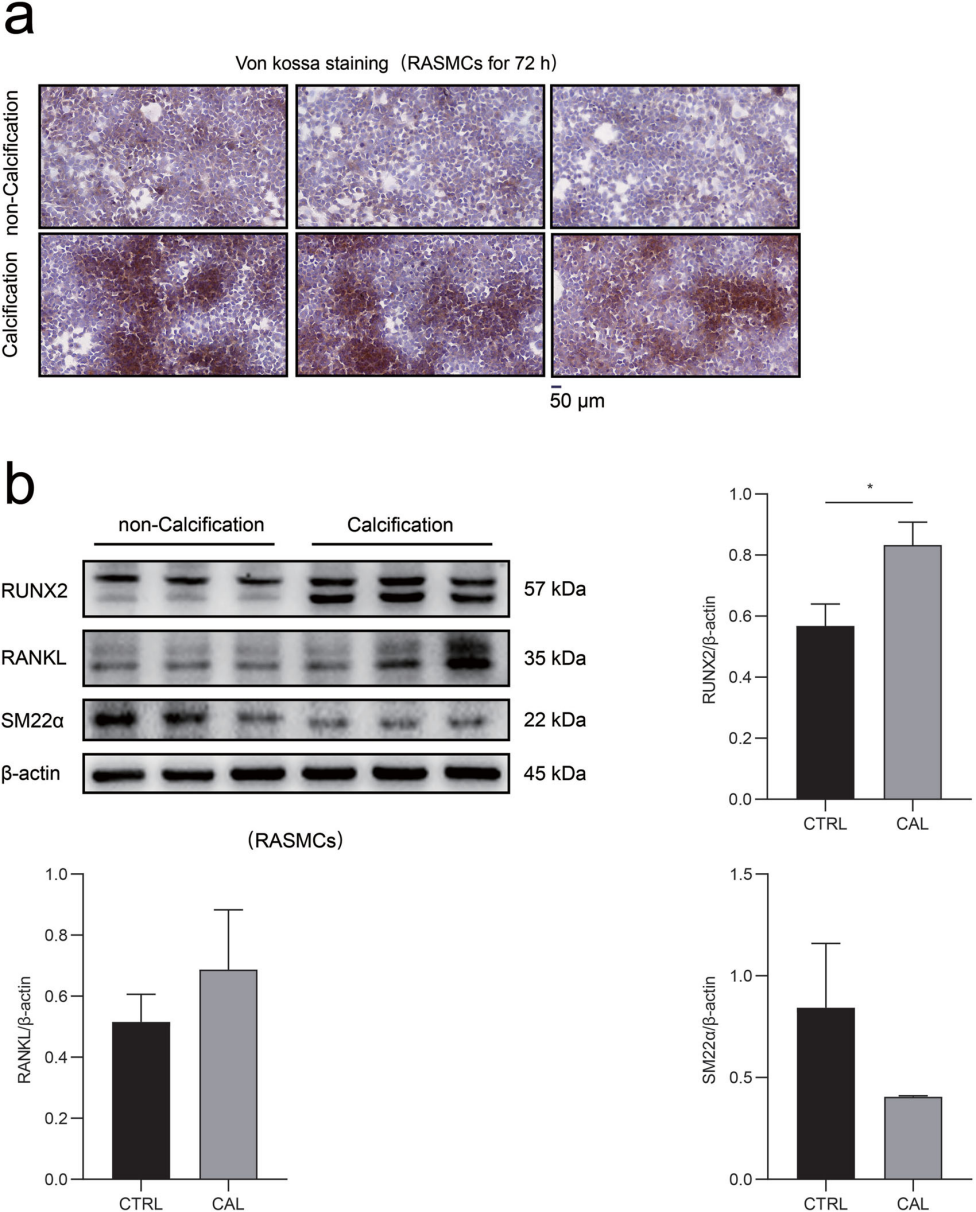
The impact of high phosphate-induced calcification in RASMCs. (a) RASMCs were treated with or without β-glycerophosphate for 72 h and von kossa stainingwas performed. The scale bar represents 50 μm. (b) The expression levels of RUNX2, RANKL and SM22α in RASMCs, as assayed by immunoblotting between the calcification group (CAL) and the non-calcification group (CTRL). The Student’s unpaired *t*-test analysis was used, *p < 0.05. *n* = 3 for each group.

The morphological results of von kossa staining revealed significant calcium deposition in RASMCs after treatment with a high dose of β-glycerophosphate treatment for 72 h (Figure 1(a)). After stimulation with β-glycerophosphate, the expression of the vascular smooth muscle cell marker SMA22α decreased significantly in RASMCs, whereas the expression of osteoblast-like cell markers, such as RUNX2 and RANKL increased dramatically in RASMCs (Figure 1(b)). These results demonstrated that high phosphorus levels facilitate calcification in RASMCs.

### LFQ identified 37 significantly expressed proteins in RASMCs following high phosphorus treatment

**Figure 2.**
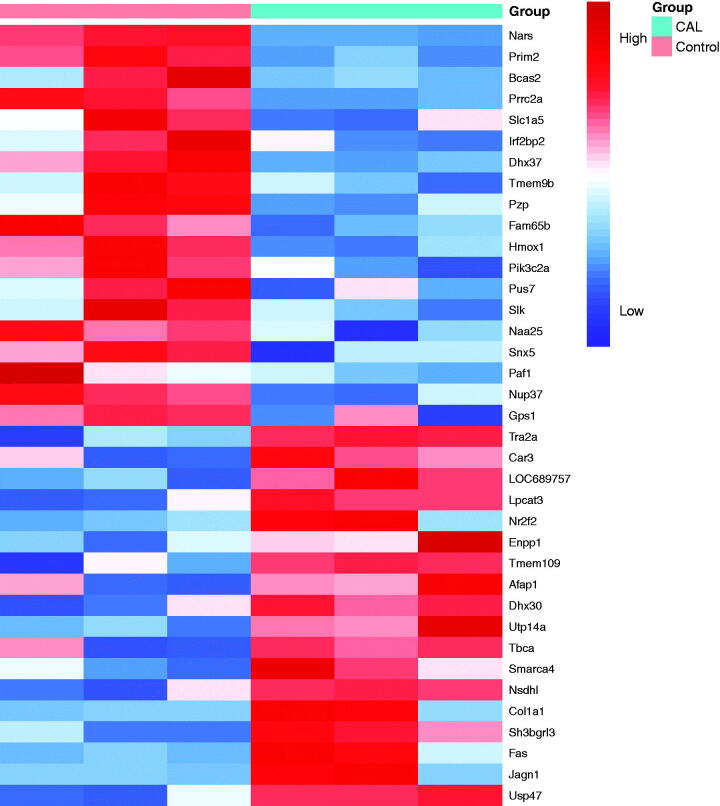
Clustering heatmap of the principal significant proteins of the LFQ intensities obtained from RASMCs during the comparison of calcification (CAL) and control groups. The experiment was performed in triplicate. The fold change of minimum was ±2 (*p* < 0.05).

LFQ analysis identified 37 proteins that were significantly expressed in RASMCs after high phosphorus-induced calcification. Eighteen proteins were upregulated and 19 proteins were downregulated in RASMCs following high phosphorus treatment. Among these proteins, the fold change for USP47 was 4.58 (*p* < 0.05), thus suggesting a significant difference between RASMCs subjected to high phosphorus-induced calcification and the normal control (Figure 2).

### USP47 increased significantly during vascular calcification

**Figure 3.**
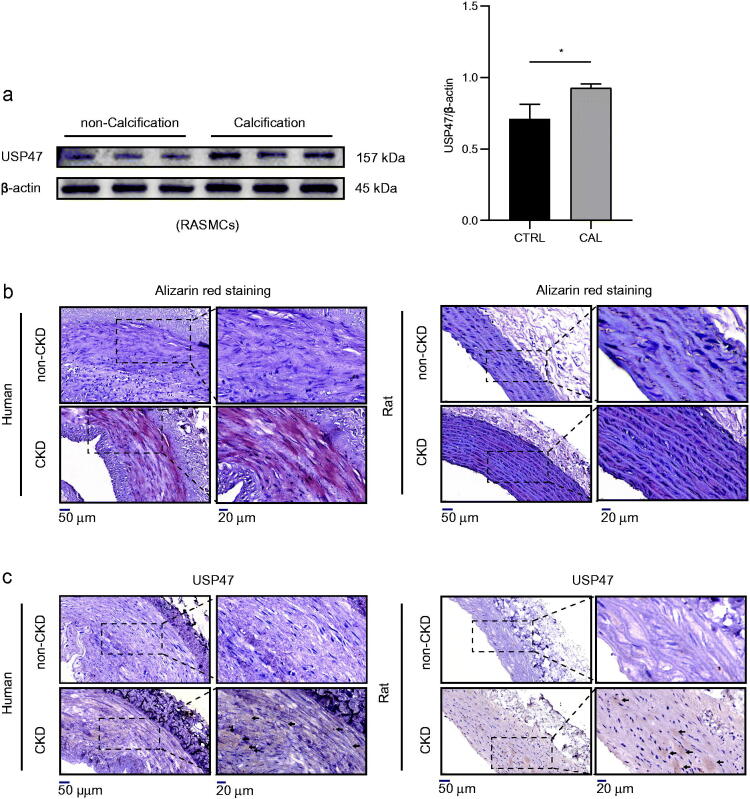
Increased expression of USP47 in CKD vascular calcification *in vivo* and *vitro*. (a) The expression levels of USP47 as assayed by immunoblotting in a high phosphorus environment for 72 h in RASMCs. The Student’s unpaired *t*-test analysis was used, *p < 0.05. *n* = 3 for each group. (b) Alizarin red staining of CKD rat abdominal aortas and CKD patient radial arteries. (c) Representative immunohistochemical staining of USP47 in aortas of CKD rats and in the radial arteries of CKD patients. Arrows mark USP47 positive areas. The scale bar corresponds to sizes from 20 to 50 μm.

In agreement with the results of the LFQ analysis in RAMSCs, we further observed the expression of USP47 in the context of vascular calcification both *in vitro* and *in vivo*. Our results revealed that USP47 expression was significantly increased in RASMCs after high phosphorus-induced calcification (Figure 3(a)). Alizarin red staining revealed increased amounts calcium deposition in both human and rat aortas after CKD vascular calcification (Figure 3(b)). Immunohistochemical staining of USP47 also showed increased USP47 expression in both human and rat aortas after CKD vascular calcification (Figure 3(c)).

### Serum USP47 increased in potential CKD vascular calcification patients

**Figure 4.**
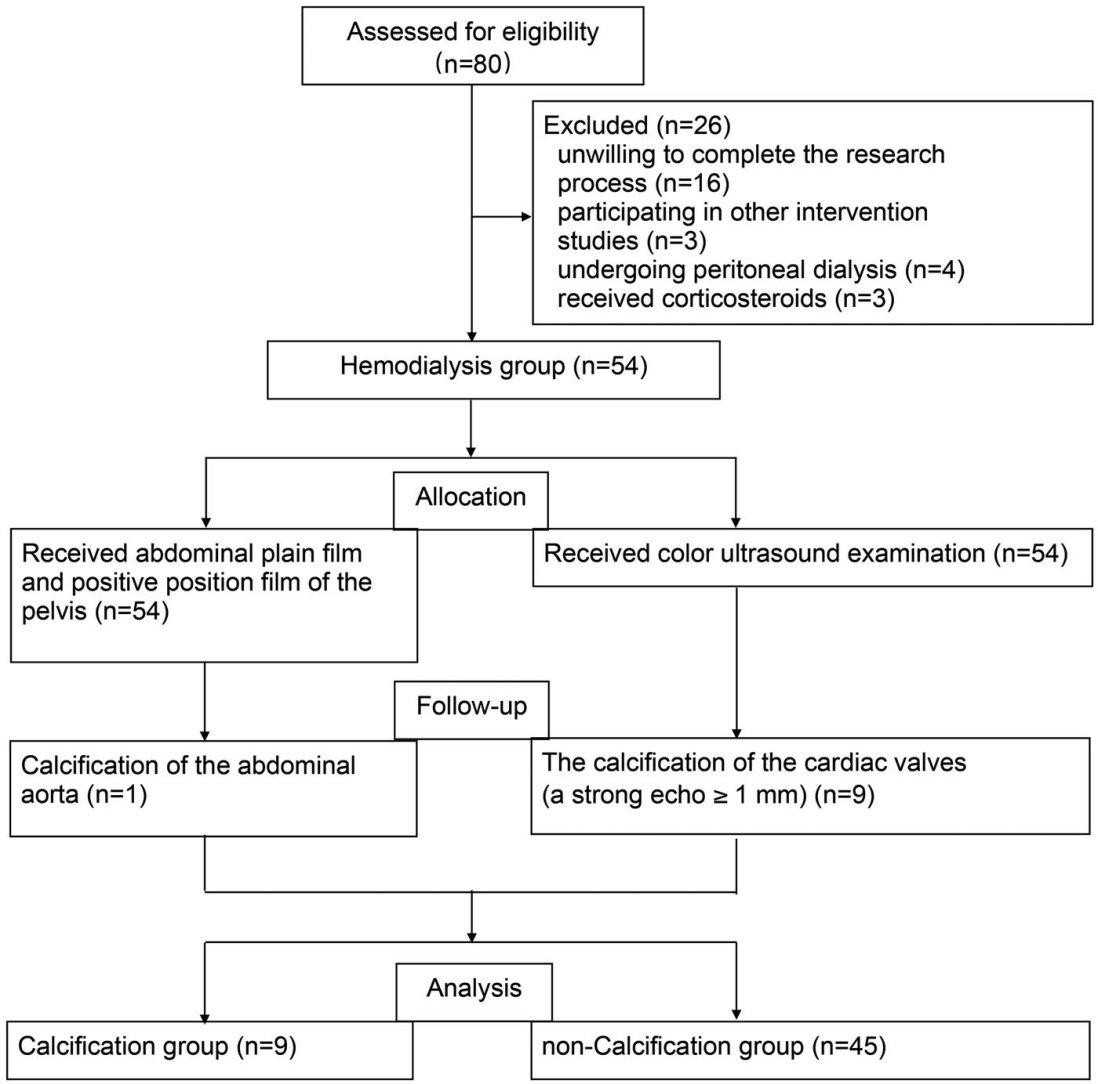
Flow chart of the study selection process.

**Figure 5.**
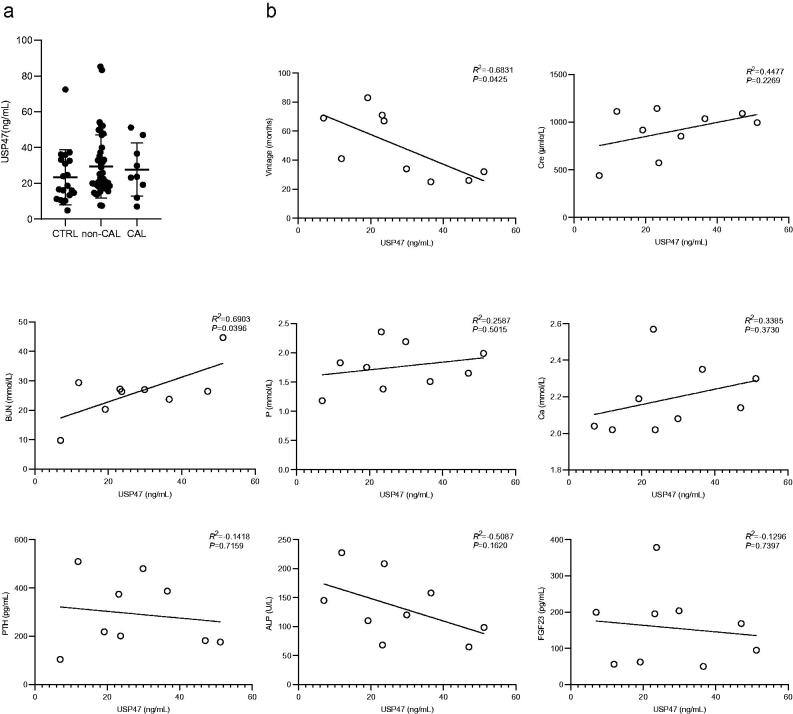
Levels of USP47 in the serum of hemodialysis patients and correlation analysis. (a) Serum USP47 level of the hemodialysis calcification group (CAL), the hemodialysis non-calcification group (non-CAL), and the negative control (CTRL). (b) Correlation analysis between USP47 and the following factors that include vintage, creatinine (Cre), blood urea nitrogen (BUN), serum phosphorus (P), serum calcium (Ca), parathyroid hormone (PTH), alkaline phosphatase (ALP), and fibroblast growth factor (FGF23). Pearson correlation coefficients and two-sided *p*-value are provided.

A total of 80 potentially eligible patients were screened from January 2018 to December 2020, of these 54 (67.5%) were eligible for the study (9 subjects in the maintenance hemodialysis calcification group and 45 subjects in the maintenance hemodialysis non-calcification group). A negative control consisting of 20 healthy individuals was included in this study (Figure 4). The baseline characteristics of the study participants are presented in Table 1. The mean age was 55.77 ± 12.78 (range 23–96) years, and 48.65% (36) of the subjects were male. The serum USP47 level was significantly higher in in hemodialysis patients with vascular calcification than that in healthy controls (Table 1 and Figure 5(a)). Correlation analysis indicated that USP47 expression was positively correlated with blood urea nitrogen (BUN) levels (linear correlation coefficient = 0.6903, *p* < 0.05) and negatively correlated with vintage (linear correlation coefficient = −0.6831, *p* < 0.05). We did not observe a significant correlation between serum USP47, and other variables involved in CKD vascular calcification, including creatinine (Cre), serum phosphorus (P), serum calcium (Ca), parathyroid hormone (PTH), alkaline phosphatase (ALP), and FGF23 (Figure 5(b)).

**Table 1.** All subjects’ baseline data (*n* = 74).

	Hemodialysis group		Control group
	CAL (*n* = 9)	non-CAL (*n* = 45)	(*n* = 20)
Age (years), mean ± SD	63 ± 5.8	52.7 ± 10.9	59.4 ± 16.4
Male, *n* (%)	4 (44.4%)	24 (53.3%)	8 (40%)
Comorbidities			
Diabetes, *n* (%)	4 (44.4%) ^#^	4 (8.9%)	N/A
Hypertension, *n* (%)	7 (77.8%)	43 (95.6%)	N/A
Serum tests			
Vintage (months), median (range)	41 (25–83)	37 (13–143)	N/A
Cre (μmol/L), median (range)	995 (441–1144)^++^	994 (416–1181)^++^	65.4 (41.1–82.9)
BUN (mmol/L), median (range)	26.4 (9.8–44.8)^++^	25 (8.8–43.2)^++^	5.9 (3.1–7.3)
P (mmol/L), median (range)	1.8 (1.2–2.4)	1.7 (0.8–3.4)	N/A
Ca (mmol/L), median (range)	2.1 (2.0–2.6)^+^	2.2 (1.4–2.7)^++^	2.5 (2.3–2.6)
PTH (pg/mL), median (range)	218 (104.1–509.3)	366 (5.6–1497)	N/A
ALP (U/L), median (range)	120 (64.8–227.4)	85 (41–174)	N/A
FGF23 (pg/mL), median (range)	168.5 (50.3–378.6)	85.4 (2.9–468.5)	N/A
USP47 (pg/mL), median (range)	23.7 (7.0–51.2)	21.1 (7.1–85.3)	17.6 (4.9–72.5)

Abbreviations: CAL: calcification; Cre: creatinine; BUN: blood urea nitrogen; P: serum phosphorus; Ca: serum calcium; PTH: parathyroid hormone; ALP: alkaline phosphatase; FGF23: fibroblast growth factor, N/A: Not available.

^#^*p* < 0.05 compared with patients of non-CAL.

^+^*p* < 0.05.

^++^*p* < 0.01 compared with patients in control group.

### USP47 knockdown impaired high phosphorus-induced calcification in RASMCs

**Figure 6.**
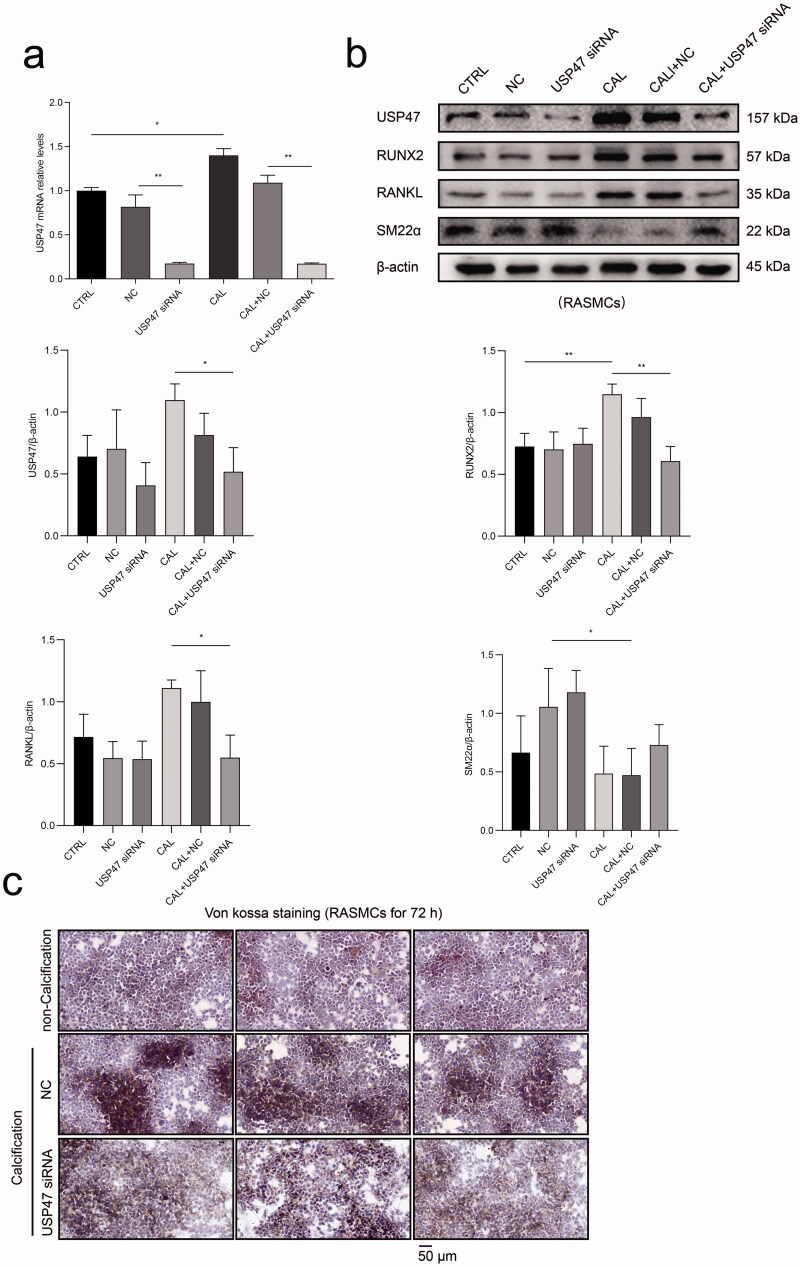
The impact of calcification with knockdown of USP47 proteins in RASMCs. (a) The level of calcification according to real-time PCR in the control group (CTRL), the negative control group (NC), and the USP47 siRNA-transfected group (USP47 siRNA) with or without calcification (CAL). One-way analysis of variance (ANOVA) was used, *p < 0.05, **p < 0.01. (b) The expression levels of USP47, RUNX2, RANKL and SM22α in USP47 siRNA RASMCs according to immunoblotting. (c) Von kossa staining of USP47 siRNA RASMCs cultured in a high phosphorus environment for 72 h. The scale bar represents 50 μm.

Real-time PCR results demonstrated that USP47 siRNA transfection effectively reduced the mRNA expression of USP47 in RASMCs. The mRNA expression of USP47 was significantly increased in RASMCs after high phosphorus treatment compared to that in RASMCs in the normal control group (Figure 6(a)). Western blot analysis confirmed that USP47 siRNA transfection effectively reduced USP47 protein expression in RASMCs. High phosphorus treatment significantly increased the expression of USP47, RUNX2, and RANKL in RASMCs, while USP47 siRNA transfection significantly reduced the expression of USP47, RUNX2, and RANKL in RASMCs. Compared with RASMCs in the normal control group, high phosphorus treated RASMCs exhibited decreased SM22α expression. However, USP47 knockdown increased the expression of SM22α in RASMCs after phosphorus treatment (Figure 6(b)). Von kossa staining indicated inhibition of calcium deposition in RASMCs after USP47 siRNA transfection with high phosphorus treatment (Figure 6(c)).

### USP47 function may involve the BTRC/AKT1 pathway in RASMCs after high phosphorus treatment

**Figure 7.**
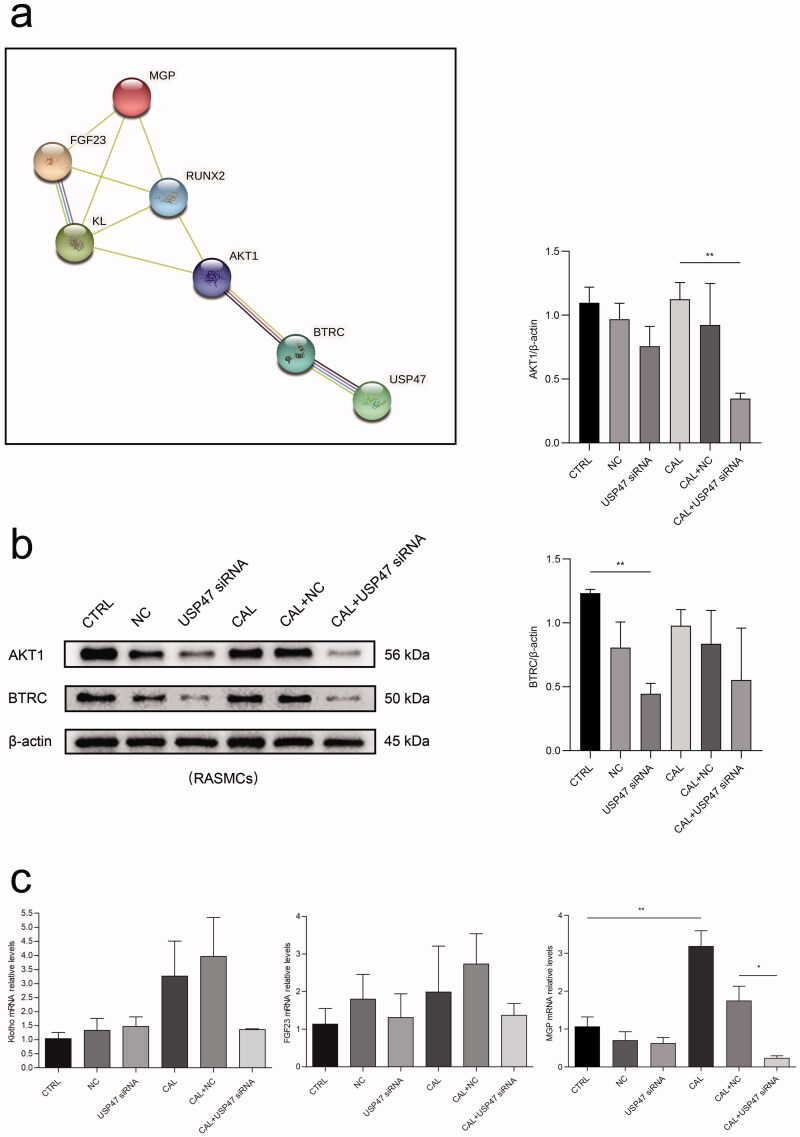
Bioinformatics analysis. (a) The protein-protein interactions network enrichment analysis. (b) The expression levels of BTRC and AKT1 in USP47 siRNA RASMCs according to immunoblotting in high phosphorus environment for 72 h. One-way analysis of variance (ANOVA) was used, *p < 0.05, **p < 0.01. (c) The levels of FGF23, Klotho (KL), and MGP in USP47 siRNA-transfected cells according to real-time PCR in high phosphorus environment for 72 h.

To identify the potential interaction between USP47 and other proteins involved in vascular calcification, we performed PPI analysis using the STRING database. USP47 may interact with the BTRC/AKT1 pathway involving RUNX2, Klotho, FGF23, and MGP upon vascular calcification (Figure 8(a)). The results of western blot analyses revealed that the USP47 knockdown significantly reduced the expression of BTRC and AKT1 in RASMCs after high phosphorus treatment (Figure 7(b)). Additionally, the USP47 knockdown inhibited the mRNA expression of Klotho, FGF23, and MGP in RASMCs following high phosphorus treatment (Figure 7(c)).

## Discussion

RASMCs were treated with a high dose of β-glycerophosphate for 72 h and it was determined that high phosphorus could facilitate calcium deposition in RASMCs, as reported in our previous study [27]. We then performed LFQ analysis on RASMCs after high phosphorus treatment. LFQ analysis identified 37 proteins that were significantly expressed in RASMCs following high phosphorus-induced calcification. Among these proteins, USP47 was expressed at a significantly higher level in RASMCs after high phosphorus-induced calcification than that in normal controls. As a member of the ubiquitin-specific protease family, USP47 plays multiple roles in cellular and pathogenic processes. USP47 regulates base excision repair, suppresses E-cadherin degradation, and facilitates NLRP3 inflammasome activation to release IL-1β and IL-18 [30–32]. USP47 also contributes to the stabilization of splicing factor IK, which is required for proper splicing of ATM pre-mRNA [33]. During oxidative stress, USP47 levels decrease and 26S proteasome activity is inhibited to reduce the rate of DNA damage repair by phenolic compounds [34]. USP47 also regulates β-catenin ubiquitination and degradation through Wnt signaling in both human and Drosophila cells [25,26]. Chen et al. observed an essential role for Wnt/β-catenin signaling in cardiovascular disease in patients with CKD [35]. However, the physiological functions and enzymatic properties of USP47 in the context of CKD vascular calcification remain unclear.

In the present study, we observed that the expression of USP47 became significantly increased both *in vitro* and *in vivo* upon CKD vascular calcification. Levels of serum USP47 were significantly higher in CKD patients with vascular calcification. Serum USP47 levels positively correlated with serum BUN levels and negatively correlated with vintage. While further elucidating the role of USP47 in CKD vascular calcification, we observed that USP47 knockdown could impair calcium deposition in RASMCs after high phosphorus treatment. During high phosphorus treatment in CKD vascular calcification, the expression of osteoblast-like cell markers such as RUNX2 and RANKL in VSMCs increased significantly and the expression of vascular smooth muscle cell marker SM22α decreased markedly, thus suggesting osteogenic transdifferentiation of VSMCs in the context of CKD vascular calcification [36,37]. Our results were consistent with those of Bao et al. [37]. We observed that USP47 knockdown increased the expression of SM22α in RASMCs after high phosphorus treatment, thus indicating the potential role of USP47 in osteogenic transdifferentiation of VSMCs in CKD vascular calcification.

To elucidate the mechanism of USP47 in osteogenic transdifferentiation of VSMCs in CKD vascular calcification, we performed PPI analysis using the STRING database. These results revealed that USP47 may interact with the BTRC/AKT1 pathway and with osteogenic transdifferentiation markers, such as RUNX2, Klotho, FGF23, and MGP, upon CKD vascular calcification. BTRC, an F-box family protein, recognizes and binds to target proteins and mediates subsequent proteasomal degradation of phosphorylated target proteins for ubiquitination [38]. Li et al. reported that AKT1 can modulate BTRC-mediated Twist1 degradation and can inhibit epithelial-to-mesenchymal transition in breast cancer [39]. Moreover, Peschiaroli et al. revealed that BTRC specifically binds to USP47 to regulate cell survival [40]. We observed that USP47 knockdown significantly reduces the expression of BTRC, AKT1, Klotho, FGF23, and MGP in RASMCs after high phosphorus treatment. In agreement with the results of the PPI analysis, USP47 may mediate osteogenic transdifferentiation markers, such as RUNX2, Klotho, FGF23, and MGP, through the BTRC/AKT1 pathway upon CKD vascular calcification.

There are some limitations to this study, and several important problems remain to be solved. We established a rat model with 0.75% adenine, 6.25 mg/kg nicotine, and 3 × 10^6^ U of vitamin D3. Kidney damage in this model was demonstrated as previously described [27]. Although there were typical features of vascular calcification, this may not be a suitable model to mimic the natural pathological process of CKD with vascular calcification. In our next study, we will identify these USP47 findings in the context of vascular calcification in the rat 5/6 nephrectomy model. Due to limitations in sample size and single-center analysis, we observed upregulation with no statistical difference in serum USP47 levels in maintenance hemodialysis patients, while a positive correlation between BUN and USP47, and a negative correlation between vintage and USP47 in hemodialysis calcification patients. To further identify the predictive role of USP47 in CKD vascular calcification, we must increase the number of clinical samples and perform multi-center studies.

In conclusion, these data indicate that USP47 may be associated with vascular calcification in CKD by regulating the osteogenic differentiation of VSMCs. This protein may regulate the process of osteogenic transdifferentiation in VSMCs upon CKD vascular calcification through the BTRC/AKT1 pathway. This study provides a novel therapeutic target for the treatment of vascular calcification in patients with CKD.

## Abbreviations

AKT1: serine/threonine-protein kinase akt-1
ALP: alkaline phosphatase
ANOVA: one-way analysis of variance
BTRC: β-transducin repeat-containing protein
BUN: blood urea nitrogen
Ca: serum calcium
CKD: chronic kidney disease
CKD-MBD: CKD-mineral and bone disorder
Cre: creatinine
FGF23: fibroblast growth factor
HD: hemodialysis
LFQ: label-free quantification
MGP: matrix Gla protein
P: serum phosphorus
PPI: protein-protein interactions
PTH: parathyroid hormone
RANKL: tumor necrosis factor ligand superfamily member 11
RASMCs: rat aortic vascular smooth muscle cells
RUNX2: runt-related transcription factor 2
SD: standard deviation
SM22α: transgelin
USP47: ubiquitin-specific protease 47
VSMCs: vascular smooth muscle cells
